# Microstructural Tuning of a Laser-Cladding Layer by Means of a Mix of Commercial Inconel 625 and AISI H13 Powders

**DOI:** 10.3390/ma12030544

**Published:** 2019-02-12

**Authors:** Maider Muro, Josu Leunda, Garikoitz Artola, Carlos Soriano

**Affiliations:** 1 IK4-Azterlan, Aliendalde Auzunea n-6, 48200 Durango, Bizkaia, Spain; mmuro@azterlan.es (M.M.); gartola@azterlan.es (G.A.); 2 IK4-Tekniker, Advanced Manufacturing Technologies Unit, Polo Tecnológico de Eibar, Calle Iñaki Goenaga 5, 20600 Eibar, Gipuzkoa, Spain; carlos.soriano@tekniker.es

**Keywords:** laser cladding, Inconel 625, AISI H13, microstructure, hardness

## Abstract

The aim of this work is to evaluate the microstructural evolutions developed by mixing a corrosion-resistant and high-performance material with a high-hardness material in a coating obtained by laser-cladding technology. In this paper, five different mixtures of Inconel 625 alloy and AISI H13 steel powders have been deposited on a plate of 42CrMo4 steel using a 2.2 kW diode pumped Nd:YAG laser. The effect of adding tool steel to a Ni-based superalloy has been analyzed by the characterization of each cladded sample using optical microscopy and scanning electron microscopy (SEM). The precipitates observed in the samples have been analyzed by energy dispersive X-ray spectroscopy (EDS X-ray). SEM micrographs and EDS analysis indicate the existence of Laves phase. It has been observed that the presence of these precipitates is stabilized in a certain range of AISI H13 addition.

## 1. Introduction

Laser cladding is an additive manufacturing process where the filler material—either wire or powder—is melted along with the substrate in order to produce coatings or even 3D parts by depositing layers on top of each other until achieving the desired geometry. Laser cladding offers many advantages over conventional coating processes such as arc welding and plasma spraying. The laser-cladding technique can produce homogeneous coatings with minimal dilution, distortion, and better surface quality than traditional technologies [1].

One of the particular advantages of the laser-cladding process when working with powder as a coating material is the possibility of combining different alloys by mixing them during the process to produce coatings with the desired chemical composition or microstructure. This approach has been widely studied by numerous authors, most of them focusing on producing metal matrix composites by combining hard ceramic particles with a metallic-based (typically Ni or Co) matrix [2,3] or functionally graded coatings (FGC) [4,5,6].

There is still another application for this powder-mixing approach which has been less investigated, consisting of improving some properties of a given alloy by adding a certain amount of another different alloy to produce new phases or compounds in the coatings. For example, Uenishi et al. and Xu et al. improved the wear resistance of an aluminium alloy by mixing it with different titanium-based compounds during the laser-cladding process [7,8]. The opposite mixture was analysed by Guo et al., who improved the tribological properties of a titanium alloy by adding aluminium [9]. Alemohammad et al. mixed titanium and cobalt base powders in order to produce high-temperature-resistant and biocompatible alloys [10]. Ni–Al mixtures were investigated by Kar et al. for increasing solid solubility of reactive elements in nickel superalloys and improving their high-temperature properties [11], and also by Duraiselvam et al., who added TiC particles to a Ni–Al mixture for protecting a martensitic stainless steel against cavitation erosion [12]. In addition, Day et al. mixed a Ni-based superalloy with different Co alloys in order to improve the wear behavior of the coatings for a better protection for turbine blades [13]. Finally, different authors centered their studies on improving certain properties of Fe alloys. For example, Corbin et al. investigated the laser-cladding process for producing corrosion-resistant Fe–Al coatings [14], Choi et al. employed a Fe–Cr–C–W mixture for producing a wear-resistant coating on a steel substrate, [15] and both Pogson et al. and Beal et al. added Cu into AISI H13 coatings in order to cope with thermal conductivity issues for injection moulds [16,17].

This experimental work explores how microstructures and hardness evolve when mixing an increasing amount of AISI H13 with Inconel 625 in laser cladding.

Inconel 625 is an austenitic Ni–Cr matrix alloy with additions of Mo and Nb to increase corrosion/oxidation resistance and high-temperature strength. C content is kept low to avoid carbide formation, while AISI H13 is a tool steel designed to work against wear by taking advantage of a Cr, Mo, and V carbide dispersion in a martensitic Fe matrix.

Both materials offer different properties and are readily available as raw materials for laser cladding, and this is what makes them candidates for FGC manufacture (e.g., a corrosion-resistant layer covering a high hardness substrate on top of a tough substrate). Nevertheless, they follow antagonistic alloying strategies that lead to microstructural issues that must be accounted for.

## 2. Materials and Methods

### 2.1. Materials

**Figure 1:**
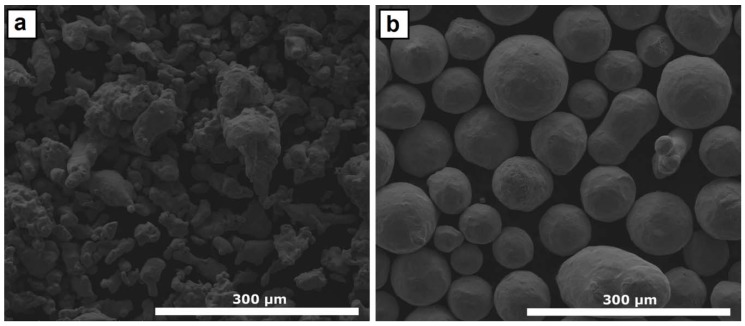
(**a**) AISI H13 and (**b**) Inconel 625 powder particles employed as raw material for manufacturing the samples.

Water-atomized AISI H13 (Figure 1a) and nitrogen-atomized Inconel 625 (Figure 1b) commercial powders from Höganäs AB (Höganäs, Sweden) and Castolin Eutectic (Lausanne, Switzerland), respectively, sieved to a range of particle sizes between 50 µm and 100 µm, were chosen as raw materials for manufacturing the samples. These two powders were placed inside different hoppers of the powder feeder in order to mix them at different ratios ranging from 10% to 50% AISI H13. The mixture was carried out by tuning the feed rates of both powders independently.

The chemical composition of the raw materials was verified by inductively coupled plasma optical emission spectrometry (ICP-OES) prior to starting the specimen manufacture (Table 1):

A plate of 42CrMo4 steel, quenched and tempered to 52 HRC, was used as the substrate for producing the coatings, with dimensions of 25 mm × 100 mm × 170 mm. The plate was grinded to a flatness of 0.01 mm.

### 2.2. Laser Cladding

A 2.2 kW diode-pumped Nd:YAG laser was used for the cladding process. The laser beam was guided to the working region by means of an optic fibre and through a coaxial cladding head. This head allows varying the laser spot on the work zone by changing the position of the focusing lens. The spot size was set to be 2.7 mm on the working plane. The powders were mixed by means of a dual powder-feeding system, with one of the hoppers filled by the AISI H13 alloy and the other one with the Inconel 625. Both powders were fed through independent pipes and combined right before introducing them in the laser head, where the mixture was produced. The laser head was fixed into a six-axis robot arm, which produced the relative motion between the laser head and the substrate. 

The process parameters were first adjusted with a powder mixture with 50 wt.% AISI H13. The main process parameters (laser power, scanning speed, and powder feed-rate) were varied until defect-free coating was achieved with minimal dilution to avoid the mixture of the alloying elements of the substrate with the coating material and a thickness of roughly 1 mm.

The parameters shown in Table 2 were employed for producing the final coating samples with different mixing proportions. A single layer of ten overlapped tracks was deposited for producing each coating.

**Figure 2:**
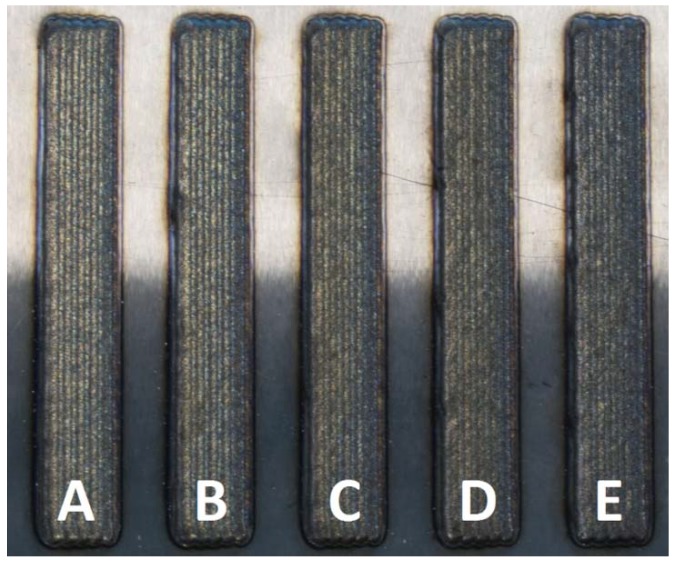
Laser-cladding samples right after being produced.

Coatings of approximately 20 × 90 mm and a thickness of 1 mm were produced onto the steel plate, separated 10 mm from each other (Figure 2). The chemical composition was checked by spark emission spectroscopy to ensure that the mixing of the powders was correctly performed (Table 3). Results confirmed that the mixing error was kept below 1% during the sample manufacture.

### 2.3. Characterization

Sample preparation sequence consisted of water cutting, followed by precision abrasive cutting to ensure that all sections were perpendicular to the plane on which the cladding layers were built. The fresh-cut surfaces were processed by SiC-based grinding and diamond, suspension-based polishing down to Ra < 0.01 µm. Samples for microstructural inspection were further processed by electrolytic etching with oxalic acid. Each sample required a slightly different condition for optimal microstructure development due to the wide range of chemical compositions involved in the study. 

The metallurgical characterization of the samples was based on optical microscopy (Leica Microsystems, Wetzlar, Germany) inspection and Vickers HV0.3 hardness (according UNE-EN ISO 6507-1) evolution mapping along the cladding layer depth. Hardness measurements were carried out on perpendicular sections (in the same cross sections used in the metallographic characterizations) of the clad zone, HAZ (heat affected zone), and substrate/base material. The hardness analyses were done using a Vickers hardness tester with a load of 0.3 kg.

In order to identify the different phases observed in the samples, the perpendicular sections of the samples were examined by a field emission gun scanning electron microscope (FEG-SEM, Ultra Pluss, Carl Zeiss AG, Oberkochen, Germany), and the nature of each phase was analysed by an energy dispersive X-ray spectroscopy (EDS, Inca Energy 350, Oxford Instruments PLC, Abingdon, UK) analysis. The EDS analyses were performed on the precipitates located in the middle of the thickness of the coating. In order to minimize the background noise, high magnification and reduced spot were employed to target the precipitates during spectra acquisition.

## 3. Results

### 3.1. Microstructure

**Figure 3:**
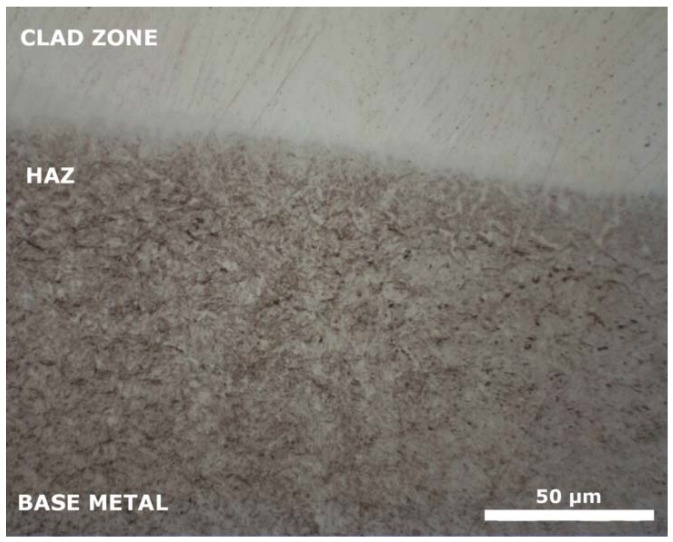
Detail of the three regions of interest in the cladding of sample A (10% H13).

Three regions of interest were identified in each coating sample, as shown in Figure 3: the clad zone, which is the top region where the material was melted; the heat affected zone (HAZ) corresponding to the region where the high temperatures of the process affected the original microstructure of the base material, and the original unaffected substrate.

In all samples, the base material was composed of a tempered martensitic microstructure. A similar HAZ was also observed in all samples, identified as bainite, judging from its hardness and morphology. 

**Figure 4:**
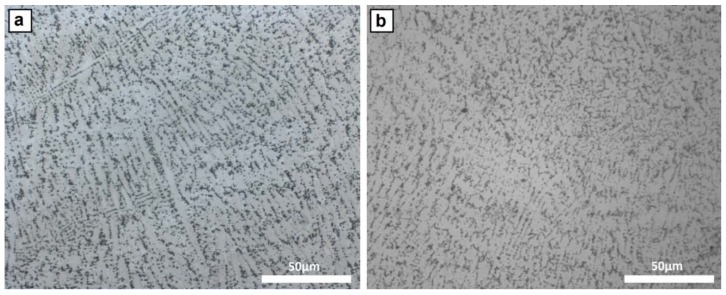
Microstructure of the clad area of (**a**) sample A and (**b**) sample E, both composed of an austenitic matrix with intermetallic precipitates.

In relation with the microstructure of the clad zone of the different coating samples, no differences were observed by optical microscopy between them. In all of the clad zones, the microstructure was composed of a nickel-based austenitic matrix with dendritic morphology and a fine distribution of intermetallic phases mainly located in the interdendritic regions, as observed in Figure 4. 

**Figure 5:**
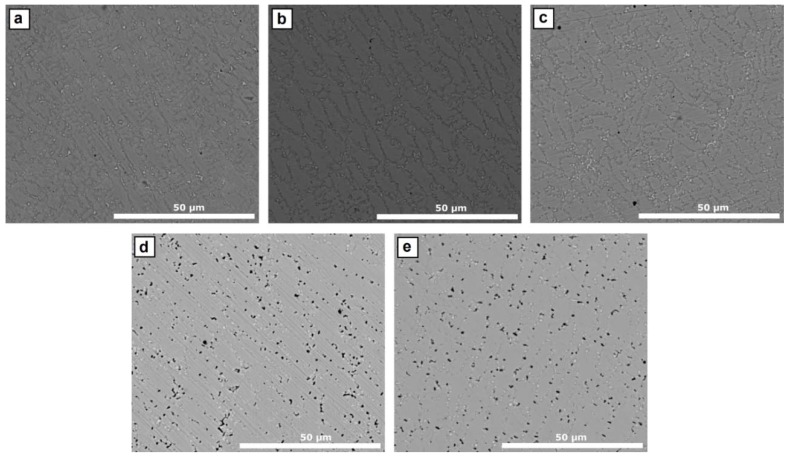
Clad-zone microstructure of (**a**) sample A, (**b**) sample B, (**c**) sample C, (**d**) sample D, and (**e**) sample E, obtained by SEM.

**Figure 6:**
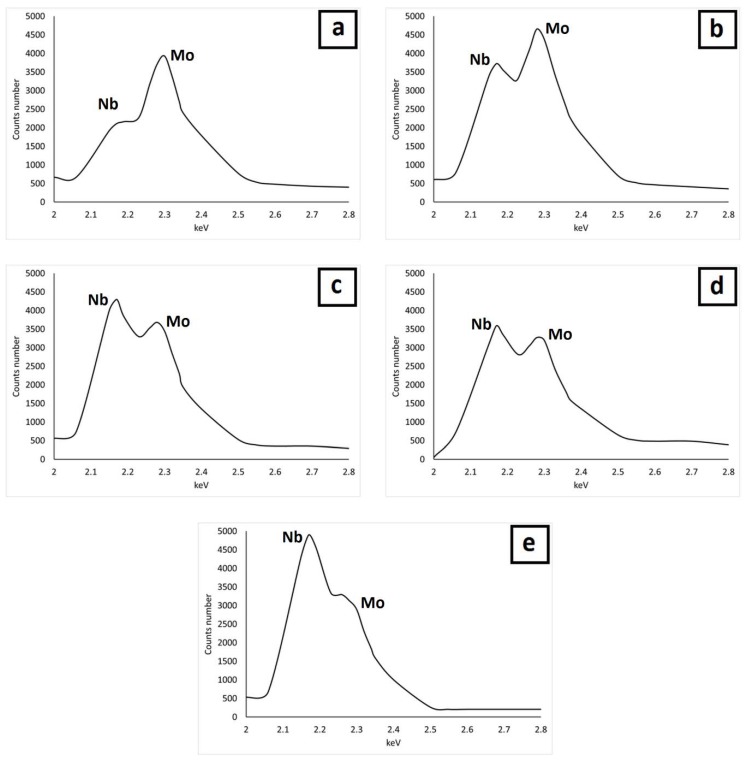
Main Mo and Nb peaks in the intermetallic phases of (**a**) sample A, (**b**) sample B, (**c**) sample C, (**d**) sample D, and (**e**) sample E, obtained by energy dispersive X-ray spectroscopy (EDS).

The microstructures examined by a back-scatter detector (BSE) under a scanning electron microscope are shown in Figure 5. All the samples show an austenitic matrix with a homogeneous dispersion of precipitates. The chemical analysis of the precipitates made by EDS allows their identification as intermetallic phases with a high content of Nb and Mo (Figure 6). Similar Nb- and Mo-rich precipitates were also observed by Jeng et al. [18], who identified them as Laves phase based on the semi-quantitative EDS results and morphology. 

**Figure 7:**
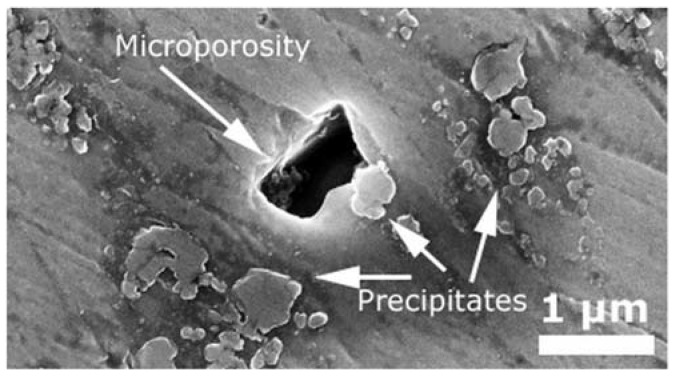
Detail of the microporosity observed in the sample E.

In the case of the samples D and E, apart from the intermetallic precipitates identified as Laves phase, black spots were observed along the whole microstructure, as shown in Figure 5d,e. These spots were identified as small pores with a size of roughly a micrometre, as can be observed in Figure 7. 

### 3.2. Hardness

Table 4 shows the average hardness values of five measurements carried out in the three specified zones: the clad zone at 0.5 mm from the surface, the HAZ at 0.8 mm from the surface, and the base metal at 20 mm from the surface.

## 4. Discussion

### 4.1. Microstructure

The intermetallic phases observed in the coatings do not precipitate when producing pure Inconel 625 coatings, according to studies carried out by other authors such as Dinda et al. [19] and Verdi et al. [20]. As-deposited structures mainly based on a γ-Ni matrix were observed by these authors, where most of the strengthening elements, like Nb and Mo, are dissolved, and carbides tend to precipitate at the interdendritic regions. It is thus clear that the mixture of this Ni alloy with a tool steel promotes the formation of the Laves phase instead of carbides. This can be explained by the inclusion of elements like Si and Fe, which strongly promote the formation of such precipitates [21].

**Figure 8:**
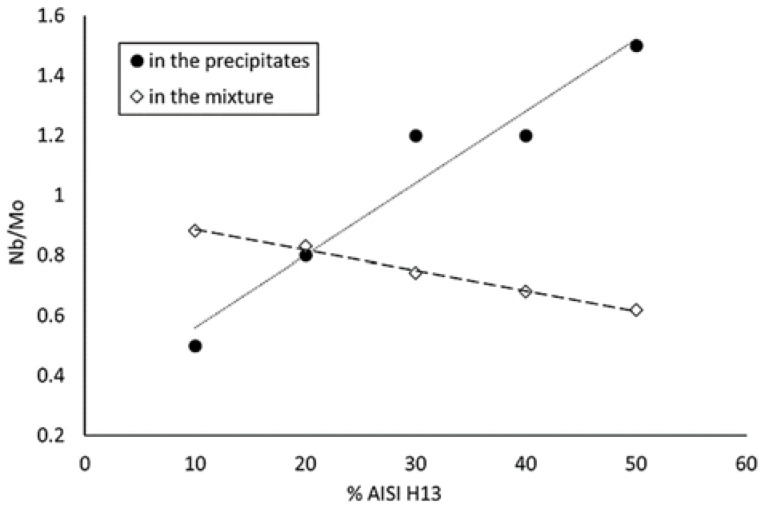
Comparison of the Nb/Mo ratio as a function of the AISI H13 content in the mixture and in the Laves precipitates.

The volume fraction of these precipitates is approximately constant up to 30% of AISI H13, despite the differences of chemical compositions between samples. This can be explained by their Nb/Mo ratio, as shown in Figure 8. Even though the total Nb-to-Mo ratio decreases in the mixture when adding AISI H13, the opposite tendency is observed in the precipitates, judging from the EDS patterns.

**Figure 9:**
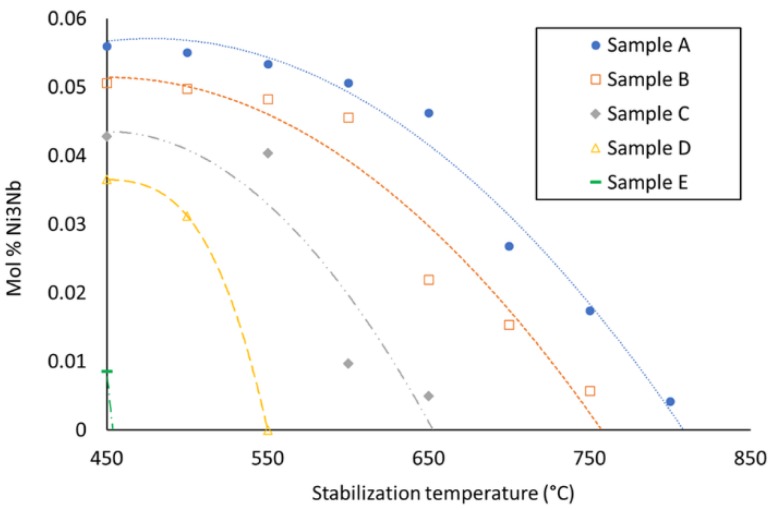
Stabilization temperature of Ni_3_Nb phase for different amounts of AISI H13.

The mechanism for keeping the volume fraction of the Laves phase constant can be explained as follows:

- According to the thermodynamic calculations by Thermo-Calc software (Solna, Sweden) using TCNI8: Ni-alloy v8.1 and MOBNI4: Ni-alloys mobility v4.0 databases/Nickel-based superalloys (TCNI8, MOBNI4) package (Figure 9) part of the Nb is combined with Ni in the submicrometric γ″ phase (Ni_3_Nb) and the rest precipitates as Laves phases together with Mo.
- As the Inconel 625 is enriched in AISI H13, the total amount of Nb + Mo in the mixture decreases.
- On the other hand, the stability of the γ″ phase from the nickel-based alloy is also reduced, thus liberating Nb, which tends to compensate the overall reduction of Nb and Mo in the precipitates.
- The amount of Nb and Mo that is actually available for forming Laves phases is thus approximately equivalent in the samples up to 30% AISI H13, but they become richer in Nb as the amount of AISI H13 increases.
- The mixture with 40 and 50% of AISI H13 steel contain less Laves precipitates, as the Nb liberated from the γ″ phase is no longer enough to compensate the overall decrease of Nb and Mo.

The porosity observed in samples D and E may be caused by the inclusion of irregular-shaped powder particles in the mixture instead of spherical ones. This kind of particle may leave small gaps between each other if they are not completely melted; thus, when the amount of AISI H13 in the mixture increases, so does the number of pores of this kind. In order to completely eliminate this kind of porosity, both powders of the mixture should be gas atomized with a spherical shape.

### 4.2. Hardness

As expected, there is no difference in the hardness values of the base steel of the different samples; in all cases, the base substrate is 42CrMo4 steel in a quenched and tempered condition with a 52 HRC hardness. The hardness of the HAZ is also equal for all samples as the process parameters were the same for every coating, and the heat affection was thus similar. Nevertheless, the hardness of the clad zone decreases as the amount of AISI H13 increases in the mixing powder. This result may result counterintuitive as the hardness of a pure H13 coating is much harder than a pure Inconel 625 one. Nevertheless, the reason for this softening is the already discussed decrease of the content of γ″ phase when adding AISI H13 to the mixture, while not enough Fe and C is still included for martensite formation.

## 5. Conclusions

Based on the results of mixing Inconel 625—a Ni-based superalloy—and AISI H13—a high-performance steel—for producing coatings by means of laser cladding, the following facts are concluded:

- Additions over 10% of AISI H13 steel stabilize the formation of Laves phases that are detrimental for corrosion resistance and mechanical response.
- The volume fraction of the Laves phase remains constant in the range of mixtures from 10% to 30% of AISI H13 steel.
- A mechanism to explain this behavior has been proposed: the Nb and Mo available for Laves phase formation remains nearly constant due to the reduction of the stability of γ″ precipitates.
- The increase in C and Fe caused by the addition of AISI H13 steel in the studied range reduces the hardness of the mixture, meaning that an FCG involving an AISI H13 to Inconel 625 can show a soft transition layer.
- The hardness of the mixtures is reduced by the inhibition of γ″ hardening precipitates produced by steel addition and the inhibition of the alloying strategy of the H13 steel (carbide formation in a martensitic matrix).

## Figures and Tables

**Table 1:** Chemical analysis of the raw powder materials.

Material	% C	% Si	% Mn	% Cr	% Mo	% Nb	% V	% Fe	% Ni
AISI H13	0.40	0.90	0.21	5.20	1.47	-	0.98	Bal.	0.12
Inconel 625	0.027	-	0.45	21.3	2.73	2.6	-	11.00	Bal.
Measurement uncertainty KI = 2	±0.01	±0.04	±0.01	±0.01	±0.05	±0.001	±0.004	±0.01	±0.01

**Table 2:** Process parameters for producing the final crack-free coating.

Process Parameters	
Power (kW)	1.5
Scanning speed (mm min^−1^)	900
Spot diameter (mm)	2.7
Powder feed rate (g min^−1^)	20
Overlap distance (mm)	1.0
Shielding gas	Ar
Shielding gas flow (l min^−1^)	20

**Table 3:** Chemical analysis of the different coatings produced by laser cladding.

Sample	%AISI H13	%C	%Si	%Mn	%Cr	%Mo	%Nb	%V	%Fe	%Ni
A theoretical	10	0.06	0.09	0.43	19.7	2.60	2.34	0.10	19.0	55.7
A		0.06	0.09	0.44	19.6	2.61	2.30	0.09	18.8	56.0
B theoretical	20	0.10	0.18	0.40	18.1	2.48	2.08	0.20	26.9	49.54
B		0.09	0.20	0.39	18.2	2.52	2.09	0.18	26.7	49.6
B theoretical	30	0.14	0.27	0.38	16.5	2.35	1.82	0.29	34.9	43.3
C		0.13	0.26	0.39	16.5	2.40	1.78	0.27	34.8	43.5
C theoretical	40	0.18	0.36	0.35	14.9	2.23	1.56	0.39	42.9	37.2
D		0.17	0.35	0.35	14.6	2.25	1.53	0.36	42.6	37.8
D theoretical	50	0.21	0.45	0.33	13.3	2.10	1.30	0.49	50.9	31.0
E		0.20	0.44	0.34	13.3	2.15	1.33	0.47	50.6	31.2
Measurement uncertainty KI = 2		±0.01	±0.04	±0.01	±0.01	±0.05	±0.001	±0.004	±0.01	±0.01

**Table 4:** Vickers hardness measurements carried out in the clad zone, HAZ and base metal for each cladding sample.

Sample	Hardness HV 0.3		
	Clad Zone	HAZ	Base Metal
A (10% H13)	290 ± 5.5	535 ± 5.5	549 ± 5.5
B (20% H13)	272 ± 5.5	505 ± 5.5	542 ± 5.5
C (30% H13)	245 ± 5.5	514 ± 5.5	555 ± 5.5
D (40% H13)	241 ± 5.5	529 ± 5.5	547 ± 5.5
E (50% H13)	230 ± 7.2	527 ± 5.5	554 ± 5.5
